# Trajectory of fine particles removal with diffusiophoresis and thermophoresis in a gas–liquid cross-flow array

**DOI:** 10.1039/c9ra04436a

**Published:** 2019-08-27

**Authors:** Zhijian Zheng, Zhong Chen, Guoxuan Xiong, Jiahua Zhu

**Affiliations:** State Key Laboratory Breeding Base of Nuclear Resources and Environment, East China University of Technology Nanchang 330013 China zhengzhijian0423@163.com; Chongqing Institute of Green and Intelligent Technology, Chinese Academy of Sciences Chongqing 400714 China; School of Chemical Engineering, Sichuan University Chengdu 610065 China

## Abstract

A gas–liquid cross-flow array (GLCA) system is proposed for fine particles (diameter between 0.1 μm and 2.5 μm, simplified as PM2.5) removal in exhaust gas, where the continuous and smooth wastewater films, providing huge specific surface area, each act as independent traps to remove PM2.5. The removal efficiency of PM2.5 is important for evaluating the performance of a GLCA, and the trajectory across the films determines the migration and ultimate fate of PM2.5. An analytical model based on a single film is developed to analyze the critical removal trajectory with diffusiophoresis (DP) and thermophoresis (TP) in the thermal boundary layer to calculate the efficiency, where the role of each force is examined. And experiments with a lab-scale GLCA are carried out with different vapor concentration and temperature gradients to verify the model. They both reveal that the removal efficiency can be increase sharply by increasing the humidity gradient between the bulk gas and film surface, while it increases slowly as temperature gradient increasing. Thus DP and TP have important effects on PM2.5 removal in the GLCA, and DP has a much more important effect than TP. A GLCA with appropriate humidity and temperature gradient can remove PM2.5 in a costly and efficient manner.

## Abstract 2

A gas–liquid cross flow array system can significantly improve PM2.5 removal efficiency without additional energy input.

## Graphical abstract

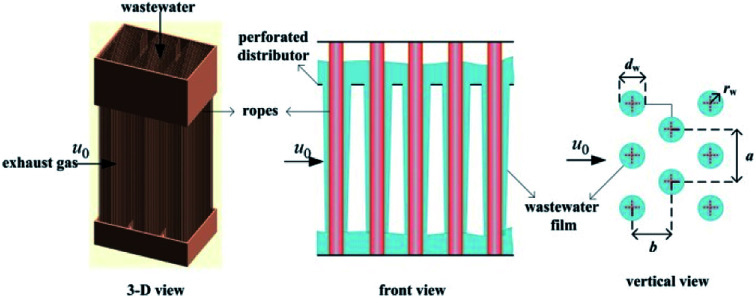

## 1 Introduction

In recent years, the removal of fine particles, emitted by coal combustion, has received great attention, since they often consist of toxic components and heavy metals, which can easily enter the human respiratory tract to create numerous health problems.^1,2^ To control the problem of particle pollution, the Chinese government has implemented the environmental standard for limiting the amount of fine particles in air, the target of which is an annual average of 35 μg m^−3^ for particles diameter *d*_p_ smaller than 2.5 μm (PM2.5).^3^ To meet this air quality limit, high efficiency electrostatic precipitators (ESPs), followed with the wet flue gas desulfurization (WFGD) systems, have been widely equipped and applied to remove particles from coal combustion,^4^ which can remove particles efficiently in the size range *d*_p_ > 2.5 μm and *d*_p_ < 0.1 μm due to inertia and interception, and Brownian diffusion respectively.^5^ Meanwhile it is still a challenge to achieve this emission standard, because particles in the size range between 0.1 μm and 2.5 μm cannot be removed effectively by ESPs and WFGD. Even worse some new aerosols (0.1 μm < *d*_p_ < 2.5 μm) are generated due to the decreasing temperature of gas in a WFGD system, which will be then emitted into air.^6^ Thus the key point to limit particles pollution is to control the emission of particles diameter in the size range 0.1 μm < *d*_p_ < 2.5 μm, and they are simplified as PM2.5 in this paper.

**Fig. 1:**
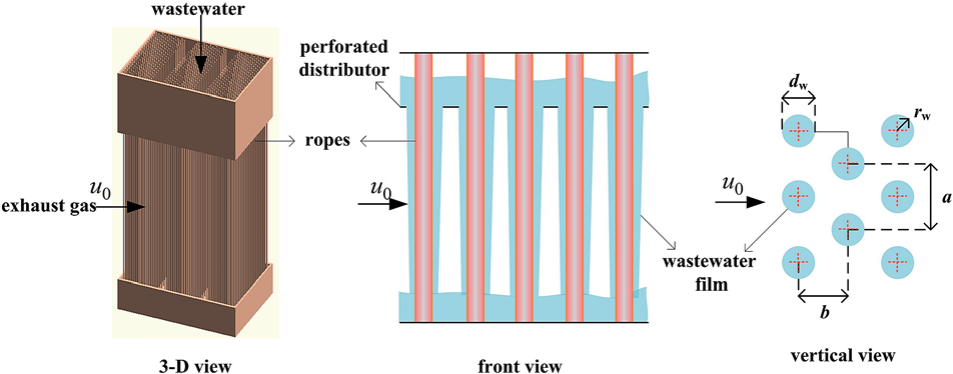
GLCA system.

A gas–liquid cross-flow array (GLCA) system is proposed for PM2.5 removal in exhaust gas from coal combustion, the three dimensional, front and vertical views of which are shown in Fig. 1.^7–9^ It is formed by numerous vertically down-flowing waste water films along numbers of triangularly configured ropes, which are installed in the middle of holes through a perforated distributor. The continuous and smooth wastewater films, providing huge specific surface area, act as independent trap each to remove PM2.5 suspending in the exhaust gas flowing perpendicularly across it.

Compared to the conventional dust collectors, a GLCA has significant advantages of self-renewable dynamic surfaces formed by cost-free wastewater without volatile contaminations and much suspended solids, which will be anyway cleaned in the downstream sewage treatment plant.^10^ And a GLCA creates besides interception, inertia and diffusion, also diffusiophoresis (DP) and thermophoresis (TP) mechanisms, which can significantly improve PM2.5 removal efficiency without additional energy input, where DP is a result of vapor concentration gradient; which cause vapor condensation onto the film surfaces, thus dragging PM2.5 along,^11^ and TP is the result of temperature gradient, which makes PM2.5 experience a net force pushing them to move in the direction of colder temperature.^12^ The vapor concentration and temperature gradients between the bulk gas and the surfaces of wastewater films are formed because exhaust gas emitted from WFGD, always approaches to be vapor saturated at about 40–60 °C, while the wastewater is at normal temperature of about 20 °C.^13^ Thus another implicit advantage of a GLCA is recovering low-grade heat and water from exhaust gas. Thereby it is promising to be taken as a cost technology for PM2.5 removal of exhaust gas by a GLCA.

The removal efficiency of PM2.5 is very important for evaluating the performance of a GLCA, and only when the mechanism of PM2.5 removal is known, and then by increasing the effect of main mechanism, the removal efficiency can be increased effectively. Zheng's work^7–9^ has developed a suitable method for the design of the GLCA and a complex model to calculate the removal efficiency, while the research about how DP and TP work and the main removal mechanism in a GLCA are still not clear. The purpose of this paper is to study the trajectory of PM2.5 with DP and TP across the water films in a GLCA to know how DP and TP work and the main removal mechanism by analysing the trajectory of PM2.5 across the wastewater films, which determines the migration and ultimate fate of PM2.5 (ref. 14–16) and then get a simple model to calculate the removal efficiency.

Because of the vapor concentration and temperature gradients between bulk gas and surfaces of films, there exists a thermal and a vapor boundary layer around it, just once PM2.5 enter into the boundary layer, they will experience the force of DP and TP, and have the possibility to be captured by the films, thus first, thickness of the boundary layer is analysed. Then the trajectory of PM2.5 in the boundary layer is studied by stress analysis, and the role of each force is examined to reveal the main removal mechanism of PM2.5. Next the model is used to calculate the PM2.5 removal efficiency of a GLCA, and experiments with a lab-scale GLCA are carried out with different vapor concentration and temperature gradients to verify the model.

## 2 Model descriptions

### 2.1 Boundary layer

**Fig. 2:**
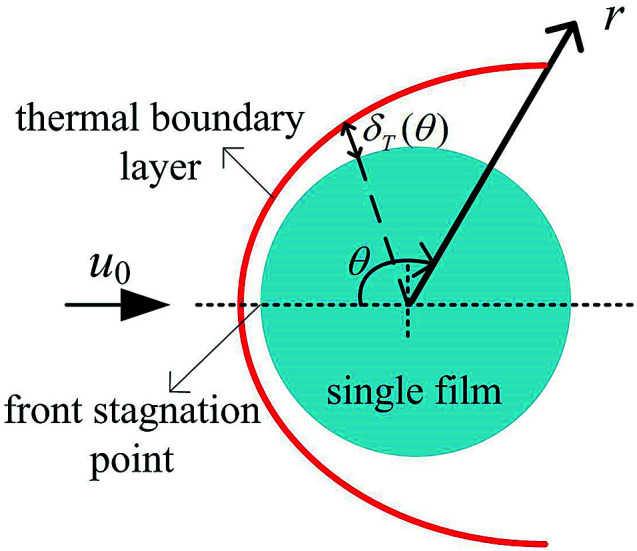
Thermal boundary layer around a single film.

Consider an incompressible dusty gas flow normal to a GLCA (far away gas velocity *u*_0_), as shown in Fig. 1, where *a* and *b* are the transverse and longitudinal pitch between each films, and *r*_w_ and *d*_w_ is the radius and diameter of a film, respectively, it will form a stable thermal and vapor condensation boundary layer around every single film due to the temperature and vapor concentration gradients. And first a model based on single film is developed to analyse thickness of the thermal boundary layer. There a cylindrical coordinate system (*r*, *θ*) fixes at the centre of the film and the angle *θ* is measured from the front stagnation point, as shown in Fig. 2.

The energy integral equation from the film surface to the thermal boundary layer outside edge with the isothermal boundary condition can be written as:^17^1
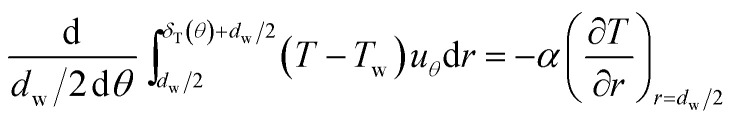


The above terms on the left and right side represent heat transfer to the film by convection and conduction respectively. *δ*_T_(*θ*) is the thickness of thermal boundary layer, *u*_*θ*_ is the gas velocity in *r* component, *α* is the thermal diffusion coefficient. In a GLCA, the volume flow rate of the recycled water is much larger than the volume flow rate of gas, thus temperature of water film *T*_w_ is assumed to be constant. And temperature of the gas in the boundary layer *T* is assumed to have a linear distribution, shown as follow:2
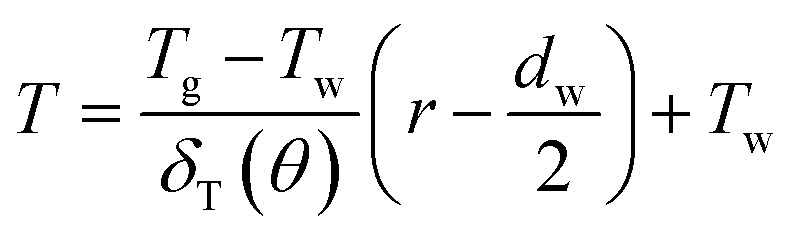
where *T*_g_ is temperature of bulk gas, which is assumed to have a constant value before every single film. Thus combined eqn (1) with eqn (2), one can obtain3
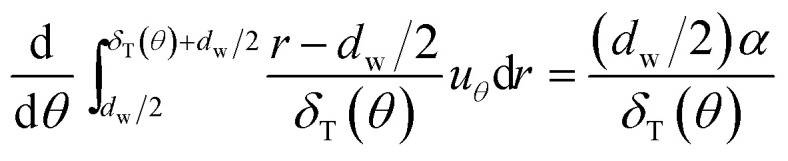


Concerned the treatment of the neighboring films interference effects in a GLCA, the Kuwabara–Happel velocity profile is used to solve eqn (3). Under very thin boundary layer condition, the stream function of the Kuwabara–Happel flow *ψ* can be approximated as flows:^18^4
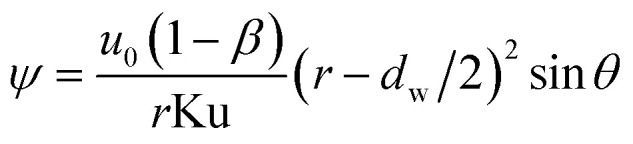


The velocity in *r* and *θ* directions (*u*_*r*_, *u*_*θ*_) can be got from the stream function in the following way:5
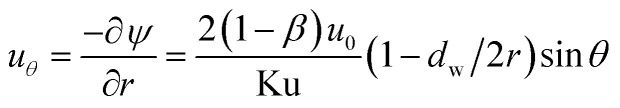
6
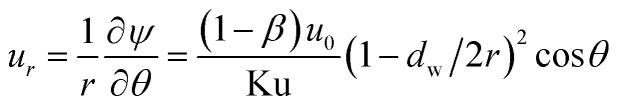
where Ku is the Kuwabara hydrodynamic factor (Ku = −1/2 ln *β* − 3/4 + *β* − 1/4*β*^2^), defined by the blockage ratio of a GLCA *β* (*β* = *d*_w_/(2*a* − *d*_w_)). Then substituting eqn (5) into eqn (3), and integrating it from the film surface at *r* = *d*_w_/2, to the outer edge of the boundary layer at *r* = *d*_w_/2 + *δ*_T_(*θ*), eqn (3) becomes:7
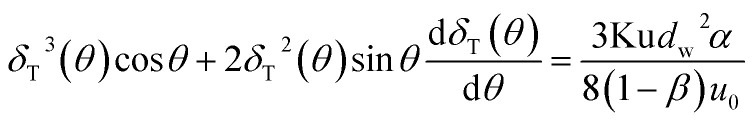


Using the assumption of thermal boundary layer thickness being 0 at *θ* = 0, thus one can obtain local boundary layer thickness:8
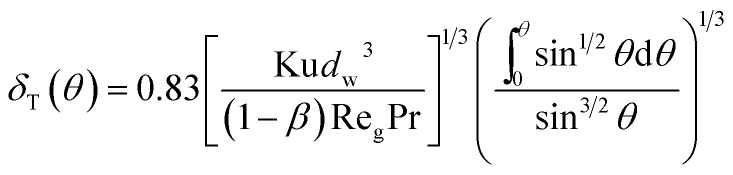
where Pr is gas Prandtl number and Re_g_ is the gas Reynolds number, which can be shown as:

9 Re_g_ = *ρ*_g_*d*_w_*u*_0_/*μ*_g_

*ρ*_g_ and *μ*_g_ are the gas density and viscosity, respectively. Simultaneously with thermal boundary layer, it also presents a thin vapor concentration boundary layer along the film. Because of low gas Reynolds number, the stream flow can be seen as laminar flow, thus the vapor concentration boundary layer can be calculated based on similarity criterion:^19^

10 *δ*(*θ*)/*δ*_T_(*θ*) = Pr^1/3^ and *δ*(*θ*)/*δ*_v_(*θ*) = Sc_v_^1/3^

where *δ*(*θ*) and *δ*_v_(*θ*) are the thickness of velocity and vapor concentration boundary layer respectively, and Sc_v_ is the Schmidt number of vapor. Thus combined with eqn (8) and (10), we can obtain the local vapor concentration boundary layer thickness:11
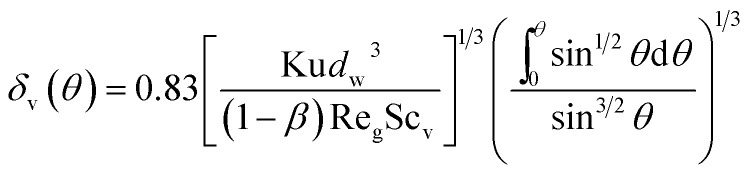


And the thermal and vapor concentration boundary layer thickness are almost equal to its value, because of the almost same value of the gas Prandtl number and the vapor Schmidt number.^20^ It means that PM2.5 enter into thermal and vapor concentration boundary layer at the same time, which can greatly simplify the model description, then just one boundary layer will be considered in the following analysis.

### 2.2 Trajectory of PM2.5 through a boundary layer with DP and TP

**Fig. 3:**
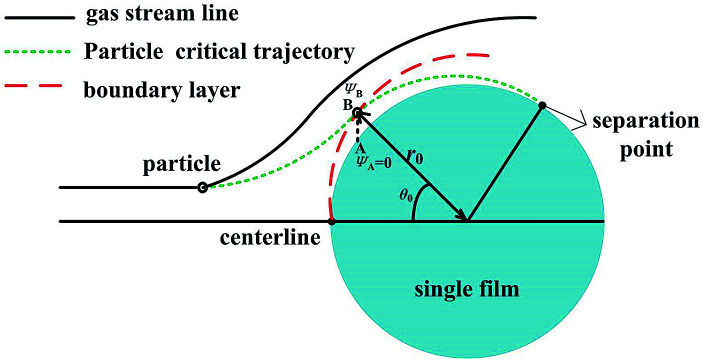
Schematic of the model used for analysing particle trajectory.

The trajectory is vitally important to evaluate PM2.5 will be captured or not. Some of PM2.5 will enter the boundary layer, which have the possibility to be captured by the films, and some will not, of course, which can't be captured. Thus our work is focused on PM2.5 entering the boundary layer. Once these PM2.5 move into the layer, DP and TP act radially onto them towards to film surface, they will have sharply different streamlines as the gas. Because vortex shedding phenomena happens with flow separation from the boundary layer, and the heat and mass transfer after the separation point can be ignored at low Reynolds numbers, if PM2.5 entering the boundary layer cannot be removed by the film before the separation point, they will escape,^21^ thus there exists a critical PM2.5 trajectory that just grazes the film surface on the boundary layer separation point, as shown in Fig. 3. It means that between centreline of the film and the critical trajectory, all PM2.5 can be removed by the film before the separation point, or PM2.5 will escape. Therefore the research can be simplified to get the critical trajectory of PM2.5 with DP and TP, as the gas flow across the films through the boundary layer in a GLCA.

Because particles discussed in this work are in the size range 0.1–2.5 μm, Brownian force is much less than the forces of DP and TP for them, which can be ignored.^22,23^ Particle growth by heterogeneous condensation can be also ignored, because a critical degree of supersaturation of vapor is not achieved.^24^ Thus non-dimensional motion equations of PM2.5 with DP and TP in the boundary layer with *R* and *θ* components are:^25^12
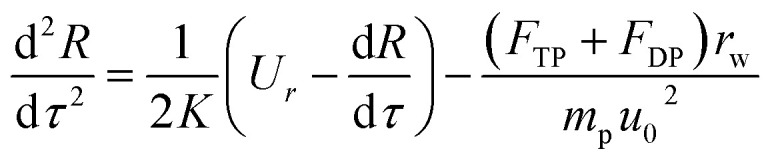
13
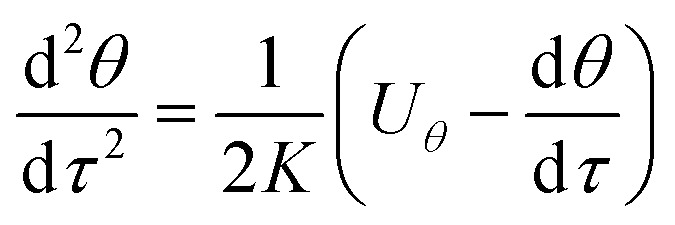
where *R*(*r*/*r*_w_) is non-dimensional radius, *τ*(*tu*_0_/*r*_w_, *t* motion time) dimensionless motion time, *U*_*R*_(*u*_*r*_/*u*_0_) and *U*_*θ*_(*u*_*θ*_/*u*_0_) the dimensionless gas velocity in *R* and *θ* components which can be obtain from eqn (5) and (6), *F*_DP_ and *F*_TP_ force of DP and TP in the boundary layer, and *m*_p_ the PM2.5 mass. And *K* is the Stokes number, which is defined as follow:

14 *K* = *Cρ*_p_*u*_0_*d*_p_^2^/36*μ*_g_*r*_w_

where *ρ*_p_, is PM2.5 density and *C* is Cunningham correction coefficient, which is a function of the gas mean free path *λ* and PM2.5 diameter *d*_p_:15
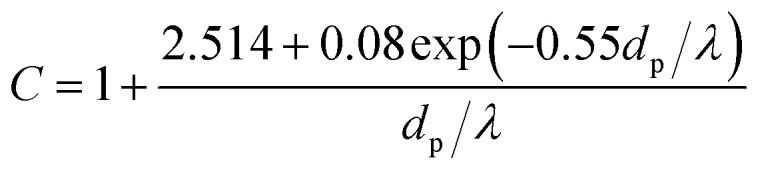


*F*_DP_ in eqn (12) is obtained by the Stokes–Cunningham equation:16
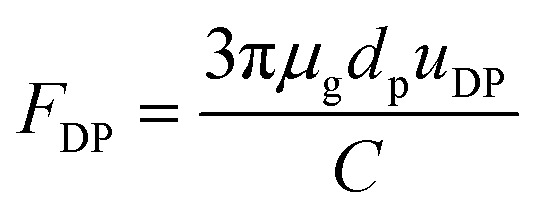
where *u*_DP_ is the diffusiophoretic velocity, which can be calculated independently of the particle diameter (once particle diameter is larger than 0.1 μm) and is given as follows:^26^

17 *u*_DP_ = −1.29*D*_v_(*H*_g_ − *H*_w_)/*δ*_v_

where *D*_v_ is vapor diffusion coefficient, *H*_g_ and *H*_w_ is absolute humidity at bulk gas and the water film surface.

Similarly, *F*_TP_ in eqn (12) is given by:18
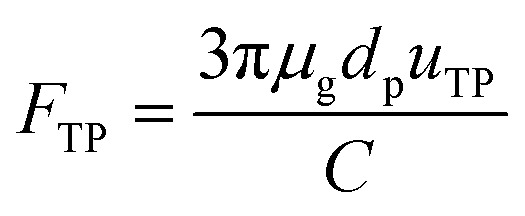


The thermophoretic velocity *u*_TP_ for particles larger than the gas mean free path is given by:^24^19
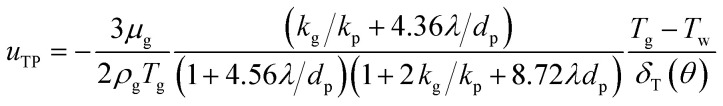
where *k*_p_ and *k*_g_ are the thermal conductivities of PM2.5 and gas, respectively.

Then by substituting the stream velocity of eqn (5) and (6), and the forces of DP in eqn (16) and (17) and TP in eqn (18) and (19) into the trajectory equations of PM2.5 in eqn (12) and (13), where the thickness of thermal boundary layer in eqn (8) needs to be substituted, we can get the analytical solutions of trajectory equations of PM2.5 with the initial conditions (*τ* = 0, *θ* = *θ*_0_ and *R* = *r*_0_/*r*_w_) as follow, which means that only once PM2.5 enter into the thermal boundary layer, then the trajectory equations of PM2.5 will just be considered.20
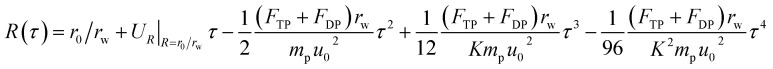
21
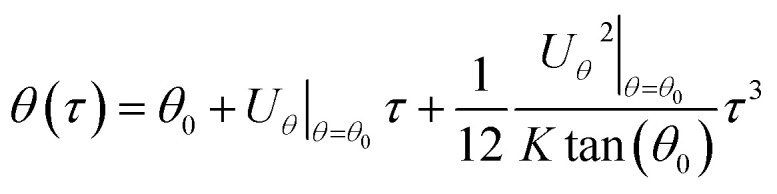


As we have described, there exists a critical PM2.5 trajectory that just grazes the film surface on the boundary layer separation point, if PM2.5 cannot be removed by the film before separation point, they will escape, then the terminal condition for eqn (20) and (21) is (*τ* = *τ*, *θ* = *θ*_s_ and *R* = 1). The separation angel *θ*_s_ was given by Ming-Hsun, shown as followed:^27^

22 *θ*_s_ = 95.7 + 267.1Re_g_^−0.5^ − 625.9Re_g_^−1^ + 1046.6Re_g_^−3/2^

Eqn (20) and (21) need to be solved by calculating the forces of DP and TP for a given particle diameter and allowing it to move for a small time interval, during which the forces are assumed to be constant, and after every time interval, the forces need to recalculate until this particle gets to separation point, then critical trajectory can be obtain, which means in turn that the initial position of point *B* for this particle (*r*_0_, *θ*_0_) in Fig. 3 can be calculated. Finally the calculating process is repeated for all the particle diameters of interest.

### 2.3 Role of each force in critical trajectory of PM2.5

To analyse the role of each force in critical trajectory of PM2.5, values of *r*_0_ and *θ*_0_ for a single film in a GLCA with different temperature and humidity gradients for a given particle diameter (*d*_p_ = 1 μm) and constant gas velocity (*u*_0_ = 0.6 m s^−1^, Re_g_ = 72.2) are calculated. The inlet gas and water parameters used in calculations are shown in Table 1. When PM2.5 just enters the thermal boundary layer, and then will be captured by the film at the separation point (the terminal condition of eqn (20) and (21)), the calculation results can draw PM2.5 critical removal trajectory.

**Table 1:** Operation parameters used for the model calculations

Gas, particle density, *ρ*_g_, *ρ*_p_/kg m^3^	1.128, 3900
Gas viscosity, *μ*_g_/Pa s	1.91 × 10^−5^
Gas, particle thermal conductivities, *k*_g_, *k*_p_/W (m K)^−1^	0.028, 3
Single film radius, *r*_w_/mm	1.05
Blockage ratio, *β*	0.25
Transverse and longitudinal pitch, *a*, *b*/mm	5.2, 4.5 mm
Water vapor diffusion coefficient, *D*_v_/cm^2^ s^−1^	0.26
Separation angle, *θ*_s_	2π/3
Kuwabara factor, Ku	0.174
Gas mean free path, *λ*/μm	0.093
Schmidt number, Sc_v_	0.7
Prandtl number, Pr	0.7

**Fig. 4:**
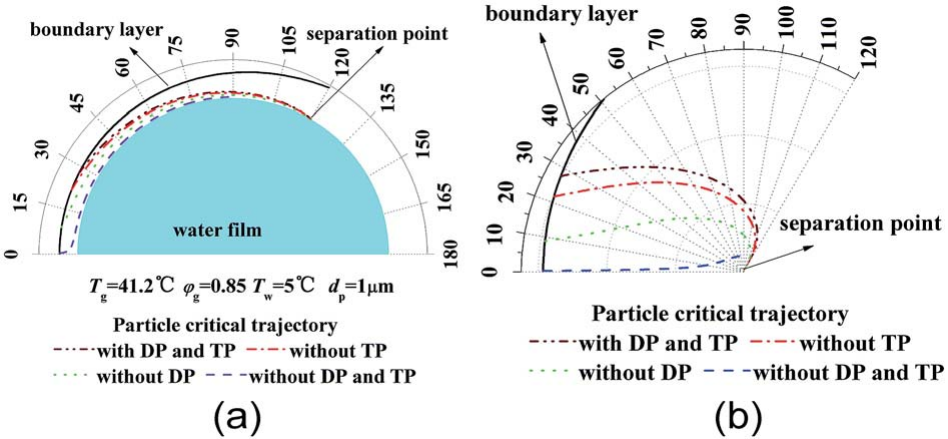
Critical removal trajectory of particle (*d*_p_ = 1 μm) in thermal boundary layer along a single film with or without DP and TP ((b) is the enlarge view of (a)).

Fig. 4 shows critical removal trajectory of particle (*d*_p_ = 1 μm) in thermal boundary layer along a single water film with different force conditions when the temperature *T*_g_ and relative humidity *φ*_g_ of gas are 41.2 °C and 0.85 respectively, and the temperature *T*_w_ of water film is 5 °C. In Fig. 4(a), the semicircle represents one single water film, where the cylindrical coordinate system (*r*, *θ*) is given to show the particle critical trajectory. Fig. 4(b) is the enlarge view of Fig. 4(a), where the water film is narrowed as a centre point to see the trajectories more clearly. First during the calculation process of eqn (20) and (21), DP and TP are supposed to be zero, which means DP and TP are not considered, the critical removal trajectory almost overlap the water film surfaces. Because the distance between the critical trajectory and the film surface represents the particle removal region, thus the PM2.5 removal efficiency is almost zero. Then during the calculation process, just DP or TP is supposed to be zero, and one of them is considered, the critical trajectories are both far away from the water film surface, and especially the distance between the critical trajectory and the film surface increases sharply when only DP is considered, which is almost double as the distance when only TP is considered. Finally, DP and TP are both considered for the calculation, the critical trajectory has a furthest distance from film surface, while the distance changes slightly as distance when only DP is considered. Thus it reveals that mechanisms of DP and TP play vitally important role for PM2.5 removal, and DP has much more important effect than TP.

To further validate it, DP and TP are both considered, while the temperature and humidity of the inlet gas are changed for the calculation. First to validate the effect of DP, eqn (20) and (21) are calculated with different humidity gradient and same temperature gradient.

**Fig. 5:**
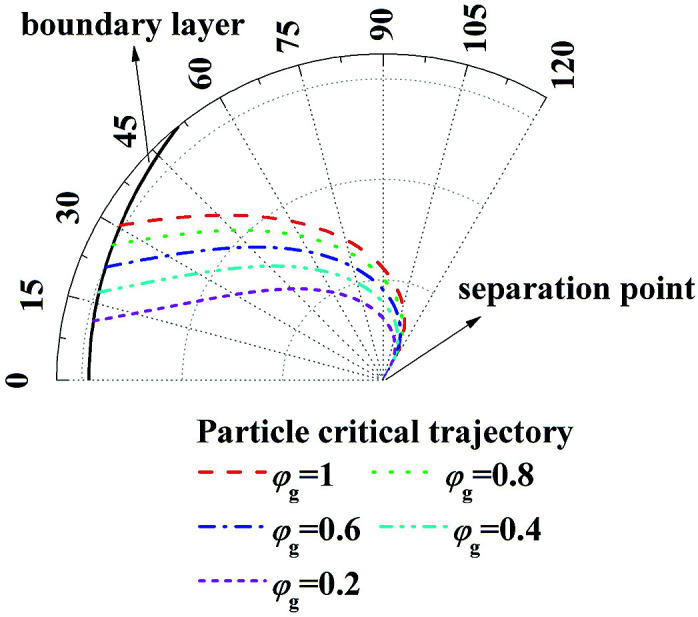
Critical removal trajectory of particle (*d*_p_ = 1 μm) in thermal boundary layer along a single film with different humidity gradient and same temperature gradient (*T*_g_ = 41.2 °C, *T*_w_ = 5 °C).

Fig. 5 illustrates the enlarge view of critical removal trajectory of particle (*d*_p_ = 1 μm) in thermal boundary layer along a single film with different humidity gradient and same temperature gradient (*T*_g_ = 41.2 °C, *T*_w_ = 5 °C). It shows, when temperature gradient between the gas and water film is constant, while as the relative humidity of gas *φ*_g_ increases from 0.2 to 1, the distance between critical trajectory and film surface increases clearly, which means the removal efficiency of PM2.5 improve heavily by increasing the effect of DP.

**Fig. 6:**
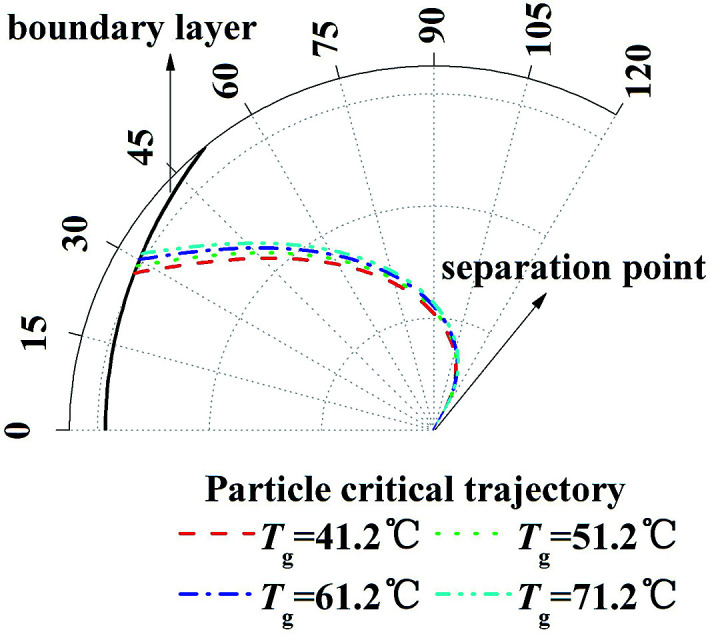
Critical removal trajectory of particle (*d*_p_ = 1 μm) in thermal boundary layer along a single film with different temperature gradient and same humidity gradient (*T*_w_ = 5 °C).

And then to validate the effect of TP, similarly, eqn (20) and (21) are calculated with different temperature gradient and same humidity gradient. Fig. 6 illustrates the enlarge view of critical removal trajectory of particle (*d*_p_ = 1 μm) with different temperature gradient and same humidity gradient (*T*_w_ = 5 °C). As the temperature of gas *T*_g_ increases from 41.2 to 71.2 °C, where the relative humidity of gas *φ*_g_ is 1 as *T*_g_ = 41.2 °C and the absolute humidity stays the same, the distance between critical trajectory and film surface just increases slightly, which means TP has a small influence on PM2.5 removal as DP.

Once the critical removal trajectory for a given particle diameter (*d*_p_ = 1 μm) was calculated, the calculations were repeated for all the particle sizes of interest.

**Fig. 7:**
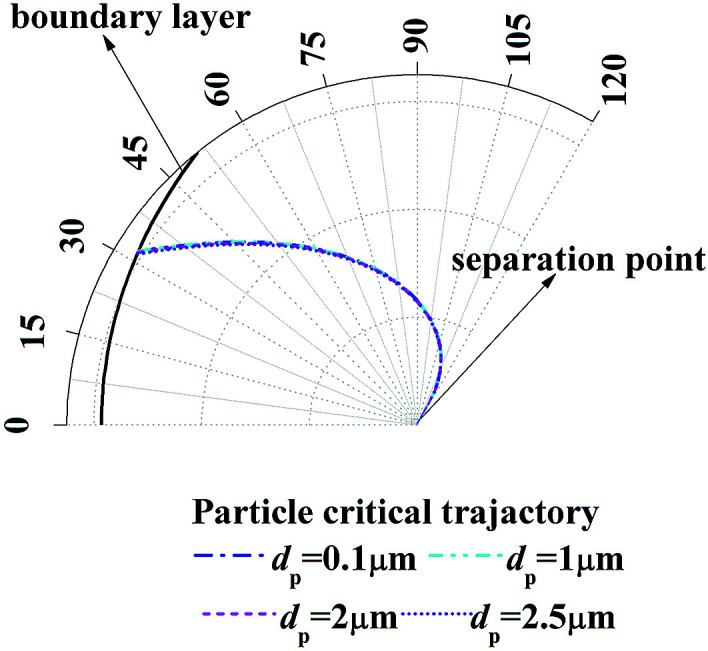
Critical removal trajectory in the thermal boundary layer along a single water film for different particle sizes (*T*_g_ = 41.2 °C, *φ*_g_ = 0.85, *T*_w_ = 5 °C).

Fig. 7 shows the enlarge view of critical removal trajectory in the thermal boundary layer along a single water film for different particle sizes when the temperature *T*_g_ and relative humidity *φ*_g_ of gas are 41.2 °C and 0.85 respectively, and the temperature *T*_w_ of water film is 5 °C. It shows that the distance between critical trajectory and film surface almost has no change when particle size changes from 0.1 μm to 2.5 μm, which is because DP is independent of particle size, and TP has a small relationship with particle size. Thus the inertial force, which has described as a function of Stokes number (the function of particle diameter shown in eqn (14)) in eqn (20) and (21), has little influence for PM2.5 removal.

### 2.4 PM2.5 removal efficiency

As described, between the critical trajectory and centreline of the film, all the particles can be removed by the film. And the initial position of point *B* for one particle has calculated with eqn (20) and (21), it is the position for one particle on the critical trajectory which just enters the thermal boundary layer. Hence PM2.5 removal efficiency by a single film *E* is defined by using the ratio of the gas volume flow rate between the critical trajectory and centreline of the film *q*_*A*–*B*_ to the total gas volume flow rate across a film *au*_0_,^28^ as shown in Fig. 3, where PM2.5 are assumed to have a uniform concentration distribution in bulk gas. And *q*_*A*–*B*_ can be obtained by stream function *ψ*, as follow:^29^23
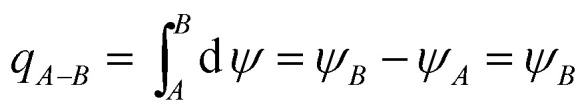
where *ψ*_*A*_ and *ψ*_*B*_ are the stream function of point *A* and *B*, and the point *A* is on the film surface, where the gas velocity is zero, thus *ψ*_*A*_ is zero. Thus PM2.5 removal efficiency by a single film *E* can be shown as:24
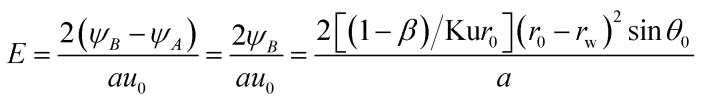


It means once values of *r*_0_ and *θ*_0_ for a single film in a GLCA are calculated with eqn (20) and (21), the removal efficiency can be got. Then the calculation of *r*_0_ and *θ*_0_ for increasing film row numbers *n* in a GLCA can be repeated for all the films one by one using the same way. Thus the efficiency by a GLCA with all the films *E*_GLCA_ can be got as flow:25
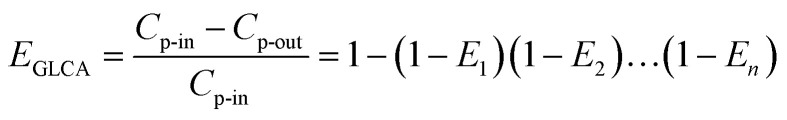
where *C*_p-in_ and *C*_p-out_ are the PM2.5 number concentration at the inlet and outlet of a GLCA, and *E*_1_, *E*_2_ and *E*_*n*_ are PM2.5 removal efficiency by a single film at the first, second and *n*-th row in a GLCA, respectively.

## 3 Experiments

**Fig. 8:**
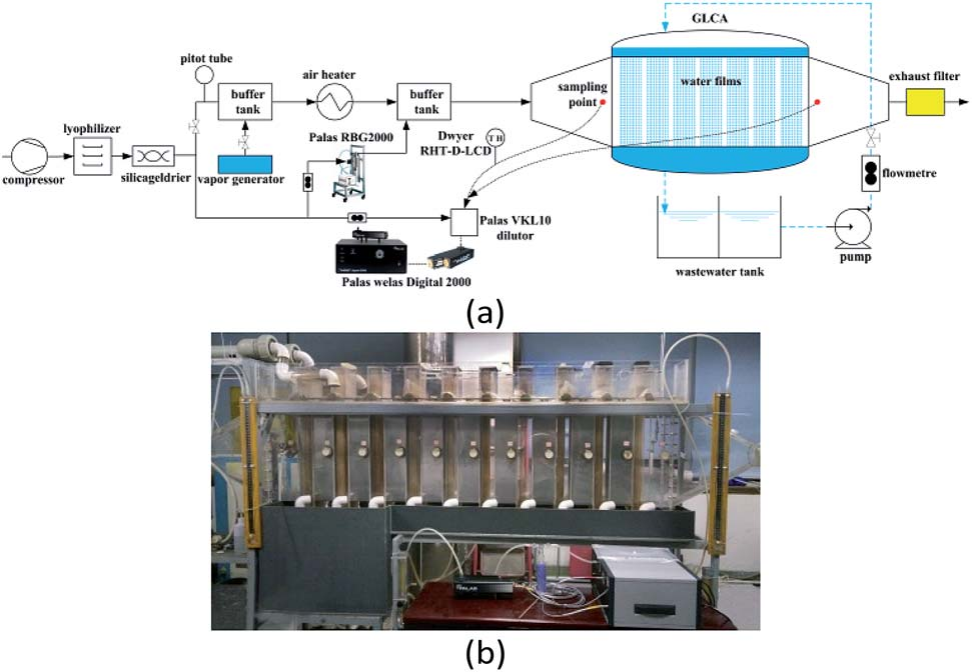
Experimental set up for PM2.5 removal: (a) flow chart of a GLCA, (b) picture of the experimental system.

The experimental setup for PM2.5 removal is shown in Fig. 8, where Fig. 8(a) is the flow chart and Fig. 8(b) is the picture of the experimental system.^8,9^ First a compressor with a silica gel drier and lyophilizer in sequence are used to provide clean gas for the system. A part of clean gas mixes with water vapor, which is generated by the vapor generator and then the mixed gas is heated by a heater, thus it can obtain the gas to be at a certain temperature and humidity. Meanwhile the other part of the clean gas proceeds across a powder dispersion generator (RBG 2000, Pales GmbH), to supply a stable PM2.5 concentration to the hot and humidity gas, thus the simulated flue gas with PM2.5 is obtained, finally it goes across the GLCA to make PM2.5 removal with DP and TP. And the essential component of PM2.5 used in the experiment is aluminium-oxide-hydroxide (Al_2_O_3_).

The structure of the GLCA is a cuboid with 60 × 12 × 80 cm in length, width, and height, respectively, which is formed by wastewater vertically down flowing along the outside edge of a mass of wires in diameter 2 mm, which are installed in the middle of the staggered arrangement holes in diameter 3.2 mm on a distribution board to form 20 water films in line and 100 water films in row respectively, and during experiment, to avoid some unnecessary interferences of wastewater, the clean water is used instead. The PM2.5 size distribution and concentration are tested at the inlet and outlet of the GLCA respectively by a Welas digital 2000 (Palas GmbH) device with an isokinetic sampling point. And also a dilutor (VKL 10, Palas GmbH) with a dilution ratio of 1 : 10 is used to avoid too much high PM2.5 concentration. And the temperature and humidity of the gas at the inlet and outlet of the GLCA are measured by Dwyer RHT-D-LCD. During every experiment, the inlet gas velocity of the GLCA *u*_0_ is 0.6 m s^−1^ (Re_g_ = 72.2) at atmospheric pressure and the volume flow rate of the recycled water is 5 m^3^ h^−1^, the inlet gas flow rate is 180 m^3^ h^−1^. And an experiment without DP and TP is done first to investigate PM2.5 removal efficiency, where the bulk gas and the water film have no temperature and humidity gradients. Then the temperature of the inlet gas and water film is constant at 41.2 °C and 5 °C respectively, and the relative humidity changes from 0.2 to 1 to measure PM2.5 removal efficiency with different humidity gradients, where the temperature gradients are constant. And then the temperature of water film is constant at 5 °C, while the gas temperature changes from 41.2 °C to 71.2 °C, and the relative humidity changes to promise that there is no humidity gradient between gas and film, which can test PM2.5 removal efficiency with different temperature gradients, where the humidity gradients are constant.

## 4 Results and discussion

**Fig. 9:**
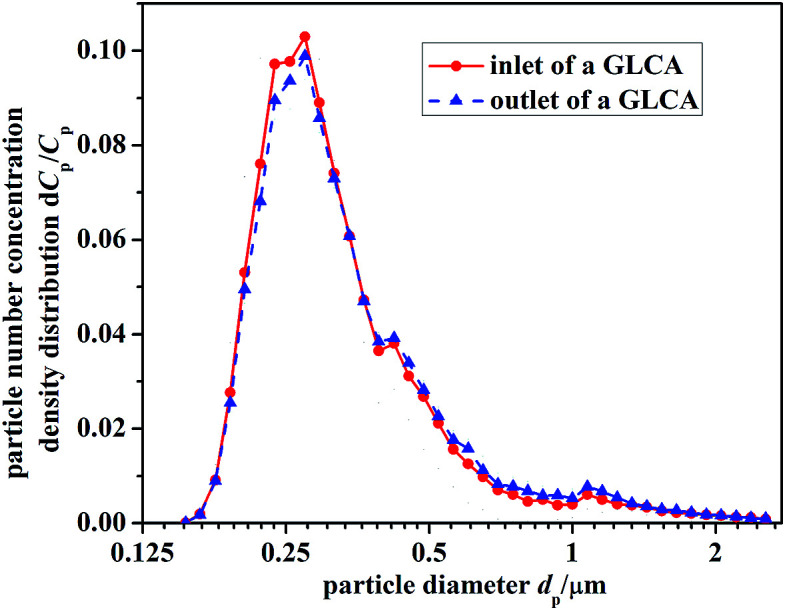
Particle number concentration density distribution d*C*_p_/*C*_p_ at the inlet and outlet of the GLCA.

Fig. 9 gives the particle number concentration density distribution d*C*_p_/*C*_p_ at the inlet and outlet of the GLCA, between which the distribution just has a slight and negligible change, thus it is reasonable to support the assumption that particle growth up due to heterogeneous condensation can be ignored. The total number concentration of PM2.5 at the inlet of a GLCA is 9690 P cm^−3^, and during every experiment it keeps stable.

**Fig. 10:**
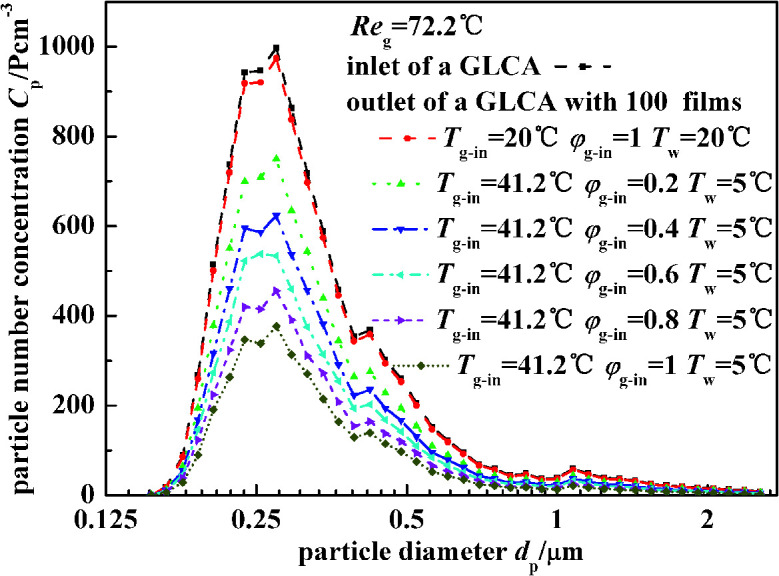
Particle number concentration at the inlet and outlet of a GLCA with 100 films in row with different humidity gradient and same temperature gradient (*T*_w_ = 5 °C, *T*_g-in_ = 41.2 °C, Re_g_ = 72.2).

Fig. 10 illustrates the experimental results of particle number concentration at the inlet and outlet of a GLCA with 100 films in row with different humidity gradient and same temperature gradient (*T*_w_ = 5 °C, *T*_g-in_ = 41.2 °C, Re_g_ = 72.2). First when the bulk gas and the water film have no temperature and humidity gradients (*T*_w_ = 20 °C, *T*_g-in_ = 20 °C), it means that there is no DP and TP, the particle number concentration between the inlet and outlet of a GLCA almost has no change. And then the inlet gas is heated and humidified to be 41.2 °C with relative humidity *φ*_g-in_ 0.2, and the water is cooled to be 5 °C, the particle number concentration decreases sharply at the outlet of a GLCA with 100 films. And to further verify the role of DP, the gas and water temperature are constant at 41.2 °C, 5 °C respectively, while the relative humidity of inlet gas increases from 0.2 to 1, particles number concentration also decreases significantly. Thus it shows DP has an important effect causing the particles removal in the GLCA for PM2.5.

**Fig. 11:**
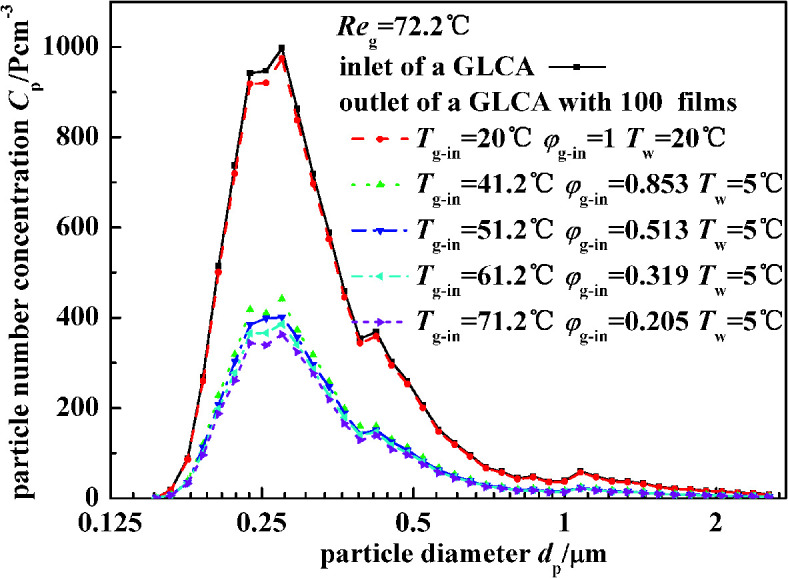
Particle number concentration at the inlet and outlet of a GLCA with 100 films in row with different temperature gradient and same humidity gradient (*T*_w_ = 5 °C, Re_g_ = 72.2).

Fig. 11 illustrates the experimental results of particle number concentration at the inlet and outlet of a GLCA with 100 films in row with different temperature gradient and same humidity (*T*_w_ = 5 °C, *T*_g-in_ = 41.2 °C, Re_g_ = 72.2). Similarly when the bulk gas and the water film have no temperature and humidity gradients (*T*_w_ = 20 °C, *T*_g-in_ = 20 °C), particles number concentration between the inlet and outlet of a GLCA almost has no change. And then the inlet gas is heated and humidified to be 41.2 °C with relative humidity *φ*_g-in_ 0.853, and the water is cooled to be 5 °C, the particle number concentration decreases sharply at the outlet of a GLCA with 100 films. And to further verify the role of TP, the water temperature is constant at 5 °C, while the gas temperature changes from 41.2 °C to 71.2 °C, and the relative humidity also changes to promise that there is no humidity gradient between gas and film, particles number concentration just decreases slightly. Thus it shows TP has a small effect causing the particles removal in the GLCA for PM2.5.

Once the particle number concentration at the inlet and outlet of a GLCA are known, the experimental result of PM2.5 removal efficiency can be calculated as follow:26
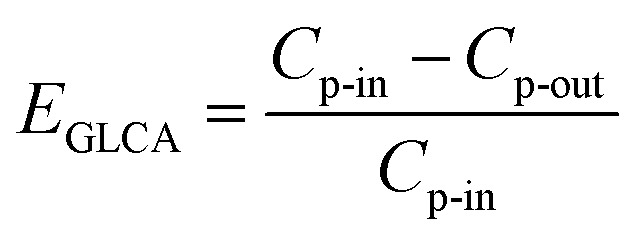


And also PM2.5 removal efficiency by a single film can by calculated using eqn (24), once the values of *r*_0_ and *θ*_0_ are calculated with eqn (20) and (21). Then the calculation of efficiency by a single film for increasing film row numbers *n* in a GLCA can be repeated for all the films one by one using the same way, the theoretical result of PM2.5 removal efficiency by a GLCA with 100 water films can be calculated using eqn (25).

**Fig. 12:**
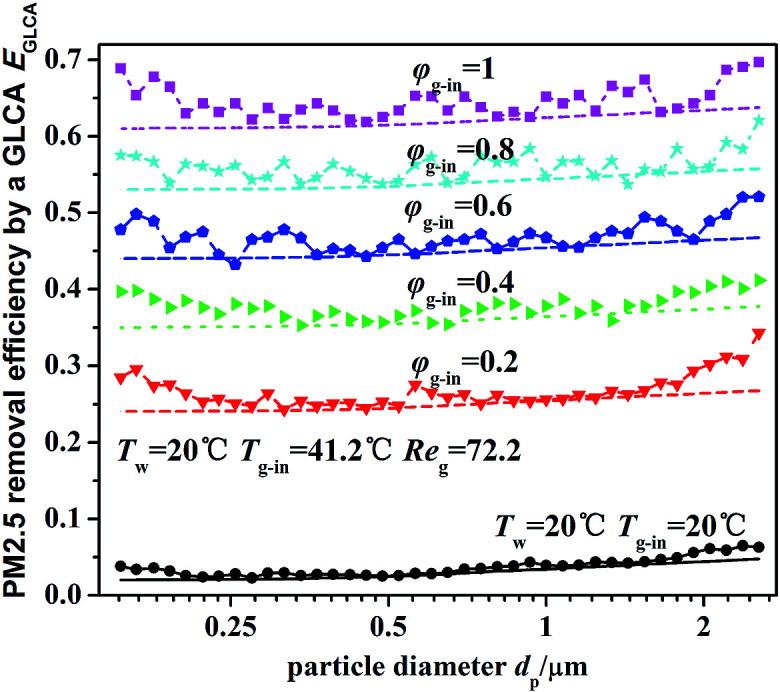
PM2.5 removal efficiency by a GLCA after 100 films with different humidity gradient and same temperature gradient (*T*_w_ = 5 °C, *T*_g-in_ = 41.2 °C, Re_g_ = 72.2).

Fig. 12 illustrates the theoretical and experimental results of PM2.5 removal efficiency by a GLCA with 100 water films as the relative humidity of inlet gas *φ*_g-in_ increasing from 0.2 to 1, when the temperature gradient keeps constant (*T*_w_ = 5 °C, *T*_g-in_ = 41.2 °C). It shows that PM2.5 removal efficiency is only about 3% at average without DP and TP, while as the relative humidity of inlet gas increases from 0.2 to 1 (*T*_g-in_ = 41.2 °C, *T*_w_ = 5 °C), the efficiency increases sharply from about 24.5% to about 63.5%. And also the efficiency increases slowly as particle diameter increase, it is because the effect of inertial impaction is still not very strong at this particle size range and DP is independent of particle diameter. And the theoretical results (lines without points) approximately consist with experimental results (lines with points), but at both ends of the experimental results, there exists a large fluctuation, which may mainly result from lack of sufficient particles, as shown in Fig. 9.

**Fig. 13:**
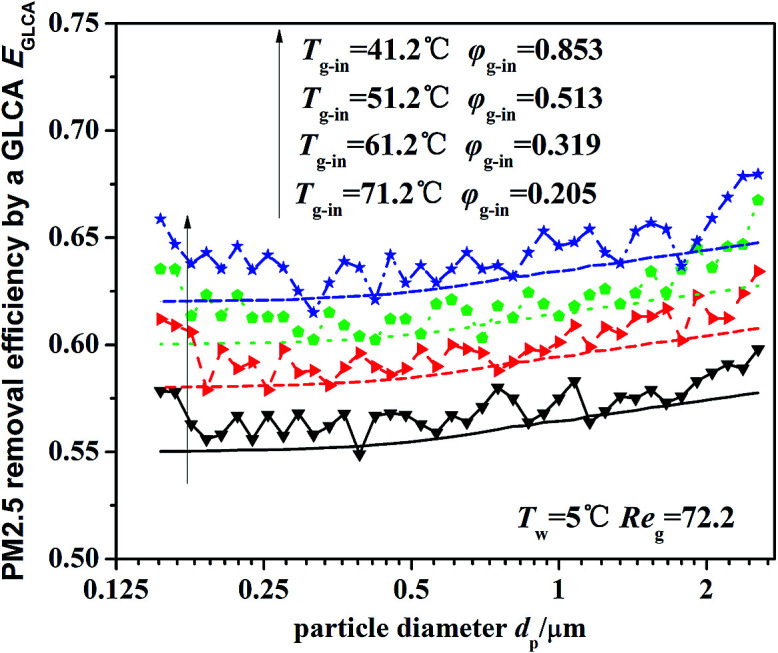
PM2.5 removal efficiency by a GLCA after 100 films with different temperature gradient and same humidity gradient (*T*_w_ = 5 °C, Re_g_ = 72.2).

Fig. 13 illustrates the theoretical and experimental results of PM2.5 removal efficiency by a GLCA with 100 water films as the temperature of inlet gas *T*_g-in_ increasing from 41.2 to 71.2, when the humidity gradient keeps constant which is solved by precisely changing the relative humidity. It shows that as the temperature gradient increase from 36.2 °C to 66.2 °C (*T*_w_ = 5 °C), the efficiency increases slowly from about 55.5% to about 63.2%. And the theoretical results (lines without points) approximately consist with experimental results (lines with points), but also at both ends of the experimental results, there exists a large fluctuation, which is also mainly resulted from lack of sufficient particles.

Thus both the experimental and theoretical results, shown in Fig. 12 and 13, indicate that DP and TP have the more important effect causing the particles removal in the GLCA for PM2.5, and by increasing humidity gradient, the PM2.5 removal efficiency by a GLCA can be improved sharply, and by increasing temperature gradient, the efficiency just improves much smaller than the result by increasing humidity gradient. Thus it shows DP and TP are the main removal mechanisms, and DP has much more important effect than TP in a GLCA.

## 5 Conclusions

A gas–liquid cross-flow array (GLCA) system is proposed for PM2.5 removal in exhaust gas. Using boundary layer theory and limiting trajectory equation of PM2.5, a theoretical model with diffusiophoresis and thermophoresis is worked out based on a single film to study critical PM2.5 trajectory, where the role of each force is a researched separately, by which PM2.5 removal efficiency of a GLCA can be evaluated. The experiments on a lab-scale test rig are carried out with different humidity and temperature gradients. The theoretical and experimental results both show that PM2.5 removal efficiency is only about 3% without DP and TP, while as the relative humidity of inlet gas increases from 0.2 to 1 with constant temperature gradient (*T*_g-in_ = 41.2 °C), the efficiency increases sharply from about 24.5% to about 63.5%, and as the temperature gradient increase from 36.2 °C to 66.2 °C (*T*_w_ = 5 °C) with constant humidity gradient, the efficiency increases slowly from about 55.5% to about 63.2%. And the efficiency increases slowly as particle diameter increases for PM2.5. It indicates that diffusiophoresis and thermophoresis have more important effect causing PM2.5 removal in the GLCA, and diffusiophoresis has much more important effect than thermophoresis. Therefore a GLCA with appropriate humidity and temperature gradient can remove PM2.5 costly and efficiently.

## Conflicts of interest

There are no conflicts to declare.

## Abbreviations

*a*, *b*: Transverse and longitudinal pitch between each film [cm]
*C*
: Cunningham correction factor [—]
*C*_p_
: Particle number concentration [P cm^−3^]
*D*_v_
: Water vapor diffusion coefficient [cm^2^ s^−1^]
*d*_p_, *d*_w_: Diameter of particle and film [cm]
*E*
: PM2.5 removal efficiency [—]
*F*_DP_, *F*_TP_: Force of diffusiophoretic and thermophoretic [g cm s^−2^]
*H*_w_, *H*_g_: Absolute humidity of the surface film and bulk gas [kg kg^−1^]
*K*
: Stokes number [—]
Ku: Kuwabara factor [—]
*k*_g_, *k*_p_: Thermal conductivity of gas and particle [W m^−1^ K^−1^]
*m*_p_
: Particle mass [g]
*n*
: Films number in a GLCA [—]
Pr: Prandtl number [—]
*q*
: Gas volume rate between critical trajectory and centreline of the film [m^3^ s^−1^]
*R*
: Dimensionless distance in the *R* direction [—]
Re_g_: Gas Reynolds number [—]
*r*
: Dimensioned distance in the *r* direction [cm]
*r*_w_
: Radius of a single film [cm]
Sc_v_: Schmidt number [—]
*T*_g_, *T*_w_: Temperature of bulk gas and film [°C]
*t*
: Particle motion time [s]
*U*_*r*_, *U*_*θ*_: Dimensionless gas velocity of in *R* and *θ* components [—]
*u*_0_
: Inlet gas velocity [m s^−1^]
*u*_*r*_, *u*_*θ*_: Gas velocity in *r* and *θ* components [m s^−1^]
*α*
: Thermal diffusion coefficient [m^2^ s^−1^]
*β*
: Blockage ration [—]
*θ*
: Angle measured from the front stagnation point [rad]
*θ*_s_
: Separation angle [rad]
*δ*
: Boundary layer thickness [cm]
*λ*
: Gas mean free path [μm]
*μ*_g_
: Gas viscosity [Pa s]
*ρ*_g_, *ρ*_p_: Density of bulk gas and particle [kg m^−3^]
*τ*
: Dimensionless motion time of particle [—]
*φ*
: Gas relative humidity [—]
*ψ*
: Stream function [m^3^ s^−1^]

## Supplementary Material
